# A naturally occurring 4-bp deletion in the intron 4 of *p53* creates a spectrum of novel p53 isoforms with anti-apoptosis function

**DOI:** 10.1093/nar/gku1359

**Published:** 2014-12-29

**Authors:** Hui Shi, Ting Tao, Delai Huang, Zhao Ou, Jun Chen, Jinrong Peng

**Affiliations:** 1 MOE Key Laboratory for Molecular Animal Nutrition, College of Animal Sciences, Zhejiang University, 866 Yu Hang Tang Road, Hangzhou 310058, China; 2 College of Life Sciences, Zhejiang University, 866 Yu Hang Tang Road, Hangzhou 310058, China

## Abstract

p53 functions as a tumor suppressor by transcriptionally regulating the expression of genes involved in controlling cell proliferation or apoptosis. p53 and its isoform Δ133p53/Δ113p53 form a negative regulation loop in that p53 activates the expression of Δ133p53/Δ113p53 while Δ133p53/Δ113p53 specifically antagonizes p53 apoptotic activity. This pathway is especially important to safeguard the process of embryogenesis because sudden activation of p53 by DNA damage signals or developmental stress is detrimental to a developing embryo. Here we report the identification of five novel p53 isoforms. p53β is generated due to alternative splicing of the intron 8 of p53 while the other four, namely, TA2p53, TA3p53, TA4p53 and TA5p53, result from the combination of alternative splicing of intron 1 (within intron 4 of the p53 gene) of the *Δ113p53* gene and a naturally occurring CATT 4 bp deletion within the alternative splicing product in zebrafish. The CATT 4 bp deletion creates four translation start codons which are in-frame to the open reading frame of Δ113p53. We also show that TAp53 shares the same promoter with Δ113p53 and functions to antagonize p53 apoptotic activity. The identification of Δ113p53/TA2/3/4/5p53 reveals a pro-survival mechanism which operates robustly during embryogenesis in response to the DNA-damage condition.

## INTRODUCTION

p53 is a pan-transcription factor that regulates diverse biological and cellular processes (1,2). p53 plays its role by regulating the expression of hundreds of genes in response to different internal or external stimuli (3). The identification of p53 isoforms marks a new era for the study of the p53 pathway and the dimension of the complexity of p53 function is further expanded by the involvement of p53 isoforms (4,5). Thirteen p53 human isoforms have been identified and these isoforms have been implicated to regulate p53 function in different ways (5,6). Human Δ133p53 and its zebrafish counterpart Δ113p53 belong to a special type of p53 isoforms whose expression is initiated by using intron 4 of the *p53* gene as its promoter (7,8). Previous studies have shown that the transcriptional expression of *Δ113p53/Δ133p53* totally depends on the full-length p53 and the function of Δ113p53/Δ133p53 protein is to antagonize the p53 apoptotic activity selectively (9–11). Δ113p53/Δ133p53 functions at least in part through its interaction with p53 (9,12). Human Δ133p53 has been found to be highly expressed in certain cancer cells (6,13–15) while the expression of the zebrafish Δ113p53 is induced by morpholino injection (16) or by mutations in genes including *def* (1) and *sec13* (17).

Digestive organ expansion factor Def is a nucleolar protein and loss-of-function of Def in the *def^hi429/hi429^* mutant results in hypoplastic digestive organs in a cell autonomous manner (7,18). The p53 pathway is activated in *def^hi429/hi429^* that in turn up-regulates the expression of *Δ113p53* (9). Recently, it has been shown that Def and Capn3 form a complex to mediate p53 degradation specifically in the nucleoli, which explains why p53 protein is accumulated in the nucleoli of *def^hi429/hi429^* mutant cells (1). Here we report the identification of five novel p53 isoforms, namely, p53β, TA2p53, TA3p53, TA4p53 and TA5p53. p53β is generated due to alternative splicing of the intron 8 of p53 while TA2/3/4/5p53 are derived from a naturally occurring 4 bp genomic deletion in the intron 1 of the *Δ113p53* gene (part of the intron 4 of the *p53* gene), which creates four new translation start codons in the product of alternative splicing of the intron 1 of the *Δ113p53* gene. We focused on studying the function of TAp53 isoforms and showed that these new isoforms function to antagonize the p53 apoptotic function in a way similar to that of Δ113p53.

## MATERIALS AND METHODS

### Zebrafish lines and maintenance

Zebrafish were raised and maintained according to the standard procedure described in ZFIN (http://zfin.org/zf_info/zfbook/zfbk.html). The *def^hi429^* mutant line was provided by Prof Nancy Hopkins at Massachusetts Institute of Technology. Two pairs of primers derived from *lacZ* and *def* were used to genotype the *def^hi429^* mutant (7). The *p53^−/-^* mutant allele *tp53^M214K^* line was provided by Prof Thomas Look at Harvard Medical School. *Tg(Δ113p53:gfp)* transgenic fish was as described (9). CATT genotypes were identified by sequencing, the primer pair 5′- GGCGAACATTTGGAGGG-3′ and 5′- AAAACACCCTAATGCGTCTTCAC-3′ were used for PCR and the primer 5′- CACAGAACAATAAACTAATAACAC-3′ was used for sequencing.

### Morpholinos

Morpholinos were purchased from Gene Tools. *def*-MO, *Δ113p53*-MO^ATG^ and the human β-globin antisense morpholino (st-MO) were used as described previously (7). *TAp53*-MO^spl^ (5′-TTTAATCACACTTACATTCAAGCCT-3′) was designed to target the splice junction between exon 1 and intron 1 of the *Δ113^b^p53* transcript.

### RNA and protein analysis

Total RNA was extracted from different samples using TRIzol reagent (Invitrogen). For real-time quantitative polymerase chain reaction (qPCR), total RNA was treated with DNase I prior to reverse transcription and purified with RNeasy mini kit (Qiagen). First strand cDNA was synthesized using M-MLV Reverse Transcriptase (Invitrogen). The qPCR was performed on CFX96TM Real-Time System (Bio-Rad) using SsoFast EvaGreen Supermix (Bio-Rad) according to the manufacturer's instructions. Primer pairs used for qPCR were listed in Supplementary Table S1.

The methods used for protein extraction from zebrafish embryos are as described previously (9). Protein electrophoresis and western blot were performed according to the instructions provided by the manufacturers (19).

### TUNEL assay and embryo viability counting

*tp53^M214K^* mutant embryos injected with either p53 or Δ113p53 mRNA were harvested for Terminal deoxynucleotidyl transferase dUTP nick end labelling (TUNEL) assays at 10 houes post injection (hpi). The survival rate for each treatment was counted at 24 hpi. CATT^+/+^ or CATT^−/−^ AB embryos were treated with γ-ray at 24 hpf at a dosage of 24 gray (Gy). Embryos at 8 h post-treatment were harvested for western and the survival rate for each treatment at 5 days post-treatment was counted.

### Antibodies

The zf-p53 mouse monoclonal antibody A7-C10 was used as previously described (1). The zf-p53 N-terminal mouse monoclonal antibody 9.1 was purchased from abcam (ab77813). Rabbit monoclonal antibody (EPR1977Y) against glyceraldehyde 3-phosphate dehydrogenase (GAPDH) was from Epitomics (#2251–1), and rabbit polyclonal antibody against β-actin (#4967) was from Cell Signaling Technology.

## RESULTS

### Identification of a p53 alternative splicing isoform p53β

**Figure 1.**
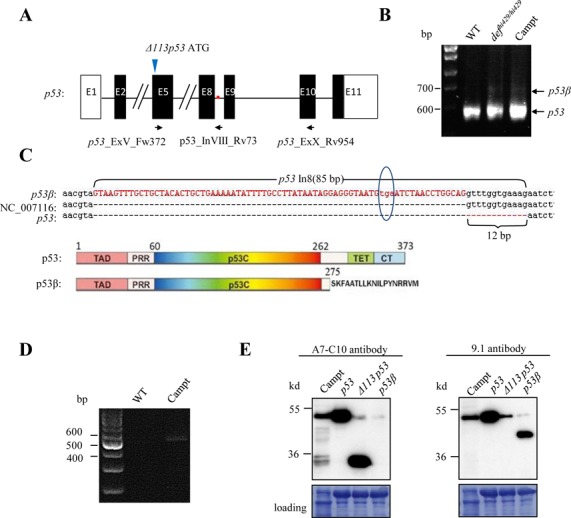
Identification of a new zebrafish p53 splicing isoform p53β. (**A**) Schematic diagram showing the genomic structure of the *p53* gene. White/black boxes, exons (E1–E11); black boxes, p53 ORF; solid line, introns; double slashes, omitted genomic region; black arrow, position and direction of primers; blue arrow head, indicating the translation start codon for *Δ113p53*. Red dot, stop codon of p53β. (**B**) Gel picture showing the RT-PCR products produced from WT, *def^hi429/hi429^* and camptothecin-treated embryos at 5 dpf using the *p53_*ExV_Fw372 and *p53_*ExX_Rv954 primers pair as indicated in (**A**). (**C**) Upper panels: alignment of full-length *p53*, NC_007116 and *p53β* cDNA sequences. 85 bp of origin of intron 8 in *p53β* was shown and this 85 bp introduces an early stop codon TGA in *p53β*. NC_007116 has an extra 12 bp of intron 8 origin when compared with the full-length *p53*. Lower panels: schematic diagram for comparison of major protein domains between p53 and p53β. (**D**) Gel picture showing RT-PCR products obtained from WT and camptothecin-treated embryos at 24 hpf using primer pair p53_ExV_Fw372 (derived from exon 5) and p53_InVIII_Rv73 (derived from intron 8). (**E**) Western blot analysis using two different monoclonal antibodies A7-C10 (left panel) and 9.1 (right panel) that specifically recognizes zebrafish p53 proteins, to assess the levels of the p53, p53β and Δ113p53 isoforms after mRNA injection. mRNA was injected into embryos at one cell stage and total protein was extracted at 6 hpf. Camptothecin-treated embryos was used as a control.

During the course of comparing the p53 and Δ113p53 protein levels in the *def^hi429/hi429^* mutant using a monoclonal antibody (A7-C10) specifically against the zebrafish p53, we noted that, in addition to the expected p53 and Δ113p53 bands, two extra bands, one with a molecular weight of ∼35 kD and another ∼45 kD, were detected (1). These two extra bands were also observed in the embryos treated with camptothecin (1,12). The higher extra band (45 kD) presumably corresponds to ΔN-p53 reported previously (20). To reveal the nature of the 35 kD band, we first performed an reverse transcriptase-PCR (RT-PCR) to amplify the cDNA fragment between the exon 5 and exon 10 (this region corresponds to the production of human p53β and p53γ through alternative splicing) (8) (Figure 1A and Supplementary Table S1) and sequenced the PCR product to check whether there is an alternative splicing in the 3′-end of zebrafish *p53* corresponding to the human p53β or p53γ (8). We obtained a PCR product (Figure 1B) which, when compared with the *p53* full-length cDNA, contained an additional 85 bp originated from the intron 8 of the *p53* gene due to alternative splicing (Figure 1C). This transcript is predicted to encode a peptide that retains the N-terminal 275 amino acids of p53 followed by the addition of 19 new amino acids (Figure 1C, Supplementary Figure S1). This new transcript is different from the GenBank sequence NC_007116 which contains an additional 12 bp originated from the intron 8 of p53 and has a predicted open reading frame (ORF) that is in-frame to the ORF of p53 (Figure 1C). To confirm that the new transcript is a genuine splicing product we performed an RT-PCR using a forward primer derived from exon 5 and a reverse primer from intron 8. A clear PCR band was obtained (Figure 1D). Sequencing of the PCR product revealed that it is identical to the new transcript (data not shown). The monoclonal antibody A7-C10 is known to recognize the C-terminus of the zebrafish p53, thus it was used to detect both the full-length p53 and Δ113p53 (1). In contrast, the monoclonal antibody 9.1 which recognizes the N-terminus of the zebrafish p53, was used to detect the full-length p53 but not Δ113p53 because Δ113p53 lacks its recognition motif (21). We cloned the PCR product into the expression vector and used the *in vitro* transcribed mRNA derived for injection. We found that this mRNA encoded for a product with much higher molecular weight which was detected by 9.1 but not by A7-C10 (Figure 1E). We designated this new p53 isoform as p53β. However, the expression of p53β protein is undetectable by the treatment of camptothecin (Figure 1E) or γ-ray (Supplementary Figure S2). Based on the above, we conclude that the 35 kD protein is not derived from the alternative splicing of the 3′-end (intron 8) of *p53*.

### Identification of *Δ113p53* alternative splicing transcripts

**Figure 2.**
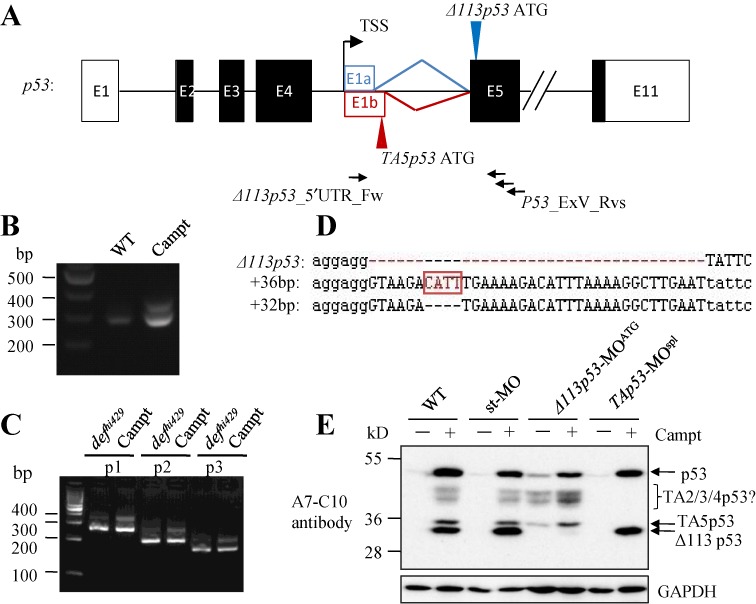
Identification of *Δ113p53* splicing transcripts. (**A**) Schematic diagram showing the genomic structure of the p53 gene. White/black boxes, exons (E1–E11); E1a box, exon 1 of *Δ113p53*; E1b, exon 1 of +36/+32 bp transcript; solid line, introns; double slashes, omitted genomic region; black arrow, position and direction of primers; blue and red arrow heads, indicating the translation start codon for Δ113p53 and TA5p53, respectively. TSS, *Δ113p53* transcription start site. (**B** and **C**) RT-PCR analysis of transcripts in WT and camptothecin-treated embryos using the *Δ113p53_*5′UTR_Fw and *p53_*ExV_Rv413 primers pair (**B**) and in *def^hi429/hi429^* and camptothecin-treated embryos using *Δ113p53_*5′UTR_Fw and *p53_*ExV_Rv413 (p1), *Δ113p53_*5′UTR_Fw and *p53_*ExV_Rv351 (p2) and *Δ113p53_*5′UTR_Fw and *p53_*ExV_Rv318 (p3) three primers pairs (**C**). (**D**) Alignment of *Δ113p53*, +36 bp and +32 bp cDNA sequences. CATT 4 bp (boxed and lettered in red) is deleted in the +32 bp transcript. (**E**) Western blot analysis of p53 and its isoforms in 36 hpf WT and camptothecin-treated embryos that were injected with either control morpholino (st-MO), *Δ113p53*-MO^ATG^ or *TAp53*-MO^spl^. Morpholinos were injected into one-cell stage zebrafish embryos. WT and injected embryos were treated with camptothecin at 24 hpf for 12 h. A7-C10, zebrafish p53 monoclonal antibody. TA2/3/4p53?, the identity of each of these bands in correspondence to TA2p53, TA3p53 and TA4p53 has not been determined. GAPDH was used as a loading control.

The transcription of *Δ113p53* is initiated in the intron 4 of the full-length *p53* gene followed by its first intron of ∼1 kb in size (7) (Figure 2A). We reckoned that the new 35 kD p53 isoform could have resulted from the alternative splicing of the intron 1 of the *Δ113p53* gene. To test this hypothesis, we designed a forward primer based on the 5′-upstream sequence of *Δ113p53* (within the intron 4 of the *p53* gene) and three reverse primers based on the exon2 of *Δ113p53* (corresponding to the exon 5 of the *p53* gene) (Supplementary Table S1). These three pairs of primers are expected to amplify part of the *Δ113p53* cDNA but not of the *p53* cDNA (Figure 2A). We first used the primer pair *Δ113p53_*5′UTR_Fw and *p53_*ExV_Rv413 (Supplementary Table S1) in RT-PCR using RNA samples obtained from the wild type (WT) and camptothecin-treated embryos. Two discrete PCR products were observed in the camptothecin-treated embryos, whereas only one faint band was detected in the WT control (Figure 2B). We then used all three primer pairs for RT-PCR using the cDNA templates obtained from *def^hi429/hi429^* mutant and camptothecin-treated embryos, respectively. Again, two discrete PCR products were observed in both *def^hi429/hi429^* and camptothecin-treated embryos (Figure 2C). We recovered the two PCR products by gel extraction for subsequent cloning. Sequencing of the clones containing the lower band revealed that it corresponds to the *Δ113p53* transcript. Surprisingly, sequencing of the higher PCR product yielded two DNA sequences that differed from a genuine *Δ113p53* sequence by addition of either 32 bp (+32 bp transcript) or 36 bp (+36bp transcript) originated from the intron 1 of the *Δ113p53* gene (Figure 2D). Sequence alignment of +36 bp and +32 bp two transcripts revealed that both transcripts are likely to be derived from the same alternative splicing product of *Δ113p53* and the difference between the +36 bp and +32 bp two transcripts is that the +32 bp transcript lacks CATT 4 bp (Figure 2D and Supplementary Figure S3).

To confirm whether +36/+32 bp transcripts resulted from alternative splicing, we designed a morpholino (*TAp53*-MO^spl^) that specifically targets the presumed splicing donor site (Supplementary Figure S3). The injection of *TAp53*-MO^spl^ abolished the expression of the 35 kD product together with other unidentified bands induced by camptothecin but did not affect on the production of Δ113p53 (Figure 2E). In contrast, the injection of *Δ113p53*-MO^ATG^, which has been shown to knock down the expression of Δ113p53 effectively (9), only blocked the expression of Δ113p53 but not the 35 kD product and other unidentified products induced by camptothecin (Figure 2E).

### The naturally occurring CATT 4bp deletion in the intron 1 of the *Δ113p53* gene is inherent in the fish population

**Figure 3.**
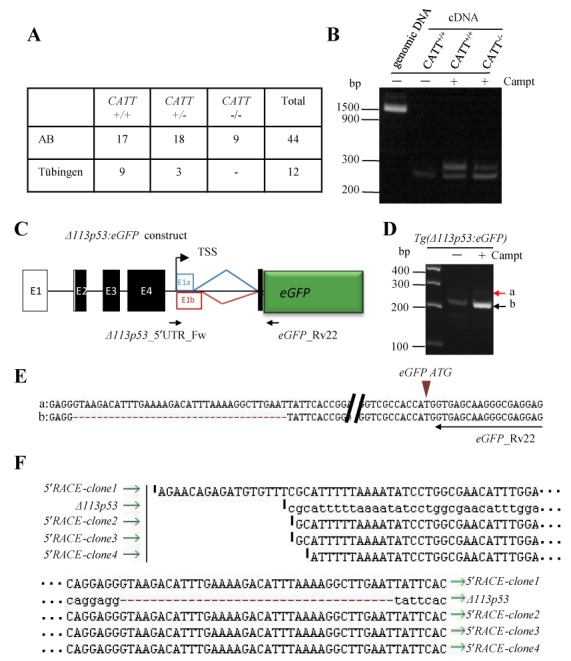
*Δ113p53* and the +36/+32 bp transcripts share the same promoter. (**A**) A CATT 4 bp deletion is naturally occurring in the intron 1 of *Δ113p53* in the existing *AB* zebrafish population. Summary of genotyping CATT^+/+^, CATT^+/−^ and CATT^−/−^ individuals in the randomly selected population. (**B**) RT-PCR analysis of +36 and/or +32 bp transcripts in embryos treated with or without camptothecin using *Δ113p53_*5′UTR_Fw and *eGFP*_Rv22 primer pairs. The same primer pair was also used in amplifying the corresponding genomic DNA fragment. (**C**) Schematic diagram showing the reporter construct in the transgenic fish *Tg(Δ113p53:eGFP)*. *Δ113p53_*5′UTR_Fw and *eGFP*_Rv22: primers pair used in RT-PCR. White/Black boxes, exons (E1–E5); E1a box, exon 1 of *Δ113p53*; E1b, exon 1 of +36/+32 transcript; TSS, *Δ113p53* transcription start site. (**D**) RT-PCR analysis of transcripts in *Tg(Δ113p53:eGFP)* embryos treated with or without camptothecin using the *Δ113p53_*5′UTR_Fw and *eGFP*_Rv22 primer pairs. (**E**) Sequence analysis of the RT-PCR product showed that the *Δ113p53:eGFP* transgene also produced a splicing transcript identical to the endogenous *Δ113p53* gene. Brown arrow head: translation start codon ATG for *eGFP*. *eGFP*_Rv22: reverse primer used in RT-PCR. (**F**) Sequence alignment analysis of the 5′-RACE products obtained from the camptothecin-treated *Tg(Δ113p53:eGFP)* transgenic fish using the *eGFP*_Rv99 and *eGFP*_Rv22 as specific reverse primers.

As stated above, the difference between the +36 and +32 bp two transcripts is the CATT 4 bp deletion in the +32 bp transcript. To find out the origin of these 4 bp deletion, we extracted genomic DNA from 44 individual *AB* line fish and amplified the DNA fragment containing this region. The PCR products were sequenced and the result revealed that 17 fish were homozygous for CATT (CATT^+/+^), 9 homozygous for CATT deletion (CATT^−/−^) and 18 heterozygotes (CATT^+/−^) (Figure 3A). In addition, 12 Tübingen adult fish were analyzed and we identified 9 CATT^+/+^ and 3 CATT^+/−^ individuals (Figure 3A). Therefore, the 4-bp deletion in +32 bp transcript is a naturally occurring deletion that is inherent in the existing *AB* fish population. We then used the primer pair *Δ113p53*_5′UTR_Fw and p53_ExV_Rv318 (Supplementary Table S1) to analyze the transcripts of *Δ113p53*, +32 bp and +36 bp in CATT^+/+^ and CATT^−/−^ fish, respectively, after camptothecin treatment. The result showed that CATT^+/+^ fish produced *Δ113p53* and +36 bp transcripts, whereas CATT^−/−^ fish produced the *Δ113p53* and +32 bp transcripts as expected (Figure 3B). In addition, we confirmed that the alternative splicing of intron 8 also occurs in +32 bp transcript by RT-PCR using a forward primer derived from the exon 1 of *Δ113p53* and a reverse primer from intron 8 of *p53* (data not shown).

### *Δ113p53* and +36/+32 bp transcripts share the same promoter

We previously reported that the *Tg(Δ113p53:eGFP)* transgenic fish which harbors the reporter gene *eGFP* underdriven by the *Δ113p53* promoter can faithfully recapitulate the response of *Δ113p53* to the developmental and DNA-damaging signals (9). Knowing that there are two genotypes for the intron 1 of *Δ113p53*, namely, CATT^+^ and CATT^−^, we first determined the genotype of the intron 1 of *Δ113p53* fused with the reporter *eGFP* and found that the transgene belongs to CATT^+^. We surmised that, if the transcription of the +36/+32 bp transcripts shares the same promoter with that of *Δ113p53*, both *Δ113p53* and the +36 bp transcript must be transcribed from the *Δ113p53:eGFP* transgene in the *Tg(Δ113p53:eGFP)* fish under the DNA damage stress. To test this hypothesis, we used a forward primer in the 5′-upstream sequence of *Δ113p53* (*Δ113p53*_5′UTR_Fw) and a reverse primer in the *eGFP* gene sequence (*eGFP*_Rv22) (Figure 3C and Supplementary Table S1) for PCR using the cDNA templates obtained from *Tg(Δ113p53:eGFP)* embryos. Clearly, two discrete PCR products were obtained in the camptothecin-treated samples, whereas only one weak band was observed in the control embryos (Figure 3D). We recovered the two PCR products by gel extraction for subsequent cloning and sequencing. The result showed that these two products represent the splicing variants of the *Δ113p53:eGFP* transgene that exactly matches the endogenous *Δ113p53* and +36 bp transcripts (Figure 3E).

We performed 5′-RACE to determine the transcription start site for the endogenous +36/+32 bp transcripts, however, due to the fact that p53 has many isoforms it was hard to identify and obtain the corresponding 5′-RACE products for the the +36/+32 bp transcripts. As an alternative, we performed 5′-RACE using the cDNA template obtained from camptothecin-treated *Tg(Δ113p53:eGFP)* embryos with a primer derived from the *eGFP* gene (Figure 3C, Supplementary Table S1). We cloned the 5′-RACE products for sequencing. Detailed analysis of the DNA sequence revealed that, the transcription start site for the +36 transcript is nearly identical to that of Δ113p53 (Figure 3F) (7). Thus, the transcription of the +36/+32 bp transcripts and *Δ113p53* was driven by the same promoter.

### TAp53 proteins arise from the CATT 4 bp deletion in the *Δ113p53* alternative splicing transcript

**Figure 4.**
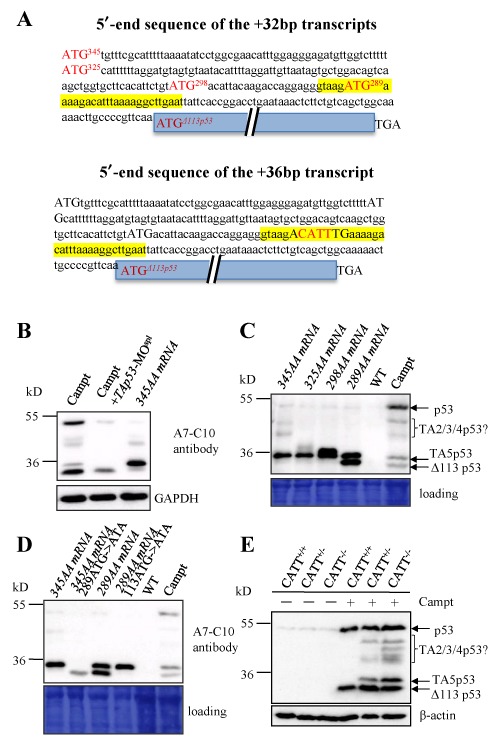
Determination of the translation start codon for the +32-bp transcript. (**A**) Upper panel: showing the 5′-leading sequence, highlighting the position of the four new ATG codons (capital letters in red) in the +32-bp transcript which are in-frame to the translation start codon ATG of Δ113p53. Lower panel: showing the 5′-leading sequence of the +36 transcript. Three ATG codons in capital letters are not in-frame to the translation start codon ATG of Δ113p53 in the +36 transcript. Solid blue bar with a double slash: *Δ113p53* ORF sequence; yellow shading: alternatively spliced sequence in +32/+36 transcripts. TGA: translation stop codon. (**B**) Western blot analysis of p53 and its isoforms in camptothecin-treated embryos injected with or without *TAp53*-MO^spl^, or in embryos injected with *345AA* mRNA. Camptothecin induced the expression of Δ113p53 and TAp53 isoforms, whereas the *TAp53*-MO^spl^ effectively knocked down the expression of TAp53 isoforms but not Δ113p53. The *345AA* mRNA expressed TA5p53 and other TAp53 isoforms. GAPDH: loading control. (**C**) Western blot analysis of protein products produced by four different mRNAs derived from the +32-bp transcripts as shown. WT and camptothecin-treated WT embryos were used as the negative and positive control, respectively. (**D**) Western blot analysis of protein products produced by different mRNAs are shown. Mutagenesis of the 289 ATG in *345AA* mRNA abolished the expression of TA5p53 (lane: 289ATG>ATA) while mutagenesis of the *Δ113p53* ATG in *289AA* mRNA abolished the expression of Δ113p53 (lane: 113ATG>ATA). In (**B**–**D**), monoclonal antibody A7-C10 was used to detect p53 and its isoforms. In (**C** and **D**), Coommassie blue staining: loading control. (**E**) Western blot analysis of p53, Δ113p53 and TAp53 isoforms in camptothecin-treated CATT^+/+^, CATT^+/−^ and CATT^−/-^ embryos, respectively, using the A7-C10 antibody. TA2/3/4p53?: the identity of each of these bands in correspondence to TA2p53, TA3p53 and TA4p53 has not been determined.

The +36-bp transcript was predicted to encode a product that is identical to that encoded by the *Δ113p53* transcript, however, the +32-bp transcript adds four new ATG codons which are in frame to the ORF of *Δ113p53* due to the CATT 4 bp deletion, leading to predicted peptide products of 345, 325, 298 and 289 AA, respectively (Figure 4A and Supplementary Figure S3). We cloned the +32-bp transcript containing coding sequence for 345 AA product (Supplementary Table S1) and obtained its mRNA by *in vitro* transcription. The +32-bp mRNA was injected into embryos and total proteins were extracted and subjected to western blot analysis. We found that the +32-bp transcript produced a major protein product of the size of 35 kD and also other protein products identical to that observed in the *def^hi429/hi429^* or camptothecin-treated embryos (Figure 4B). Thereafter, we named the new p53 isoforms derived from +32 bp mRNA as TA2p53 (corresponding to 345 AA product), TA3p53 (corresponding to 325 AA product), TA4p53 (corresponding to 298 AA product) and TA5p53 (corresponding to 289 AA product), collectively called TAp53 isoforms.

To determine whether these four new ATG codons in +32 bp transcript are genuinely used in the translation of corresponding TAp53 isoforms, we generated four constructs, namely, *345-, 325-, 298-* and *289-TAp53*, respectively, corresponding to the four new ATG codons in the +32-bp transcripts (Supplementary Table S1), for *in vitro* transcription. The mRNA corresponding to each of these four constructs was injected and total protein was extracted and subjected to western blot analysis. The result showed that the *289-TAp53* mRNA yielded two protein products identical to those observed in *def^hi429/hi429^* and camptothecin-treated embryos (Figure 4C). Injection of *298-TAp53* mRNA produced two products, one corresponding to TAp53 plus a new higher band (Figure 4C). The predominant product by *325-TAp53* mRNA injection produced TA5p53, meanwhile two faint higher bands, one corresponding to the higher band observed by *298-TAp53* mRNA injection and another being a new band (Figure 4C). Injection of *345-TAp53* mRNA also produced TA5p53 as the predominant product and a few faint higher bands (Figure 4C). Apparently, protein translation can be initiated from all four ATG codons. However, because all four mRNAs produced TA5p53 as the major product and the *289-TAp53* is the shortest one, we concluded that TA5p53 is translated by using the ATG for the 289 AA product and ATG^289^ is a preferred translation start codon.

To unequivocally prove that TA5p53 is translated from the ATG in *289-TAp53* unequivocally we mutated the 289 ATG to ATA in the *345-TAp53* transcript (Supplementary Table S1). This mutant mRNA failed to produce TA5p53 and but produced Δ113p53. When the ATG for Δ113p53 translation initiation was mutated to ATA in the *289-TAp53* transcript, the resultant mutant mRNA was no longer able to produce Δ113p53 but only produced TA5p53 (Figure 4D). These results demonstrate that the 289 ATG is responsible for the translation of TA5p53 (Supplementary Figure S4).

Next, we extracted total protein from CATT^+/+^ and CATT^−/−^ fish, respectively, after camptothecin treatment, and performed western blotting to analyze the expression of Δ113p53 and TAp53 protein in these two genotypes. We found that CATT^+/+^ fish only produced Δ113p53, whereas CATT^−/−^ fish produced Δ113p53, TA5p53 and other isoforms (Figure 4E). Therefore, these TAp53 isoforms are indeed translated into functional proteins.

### TAp53 isoforms are pro-survival factors

**Figure 5.**
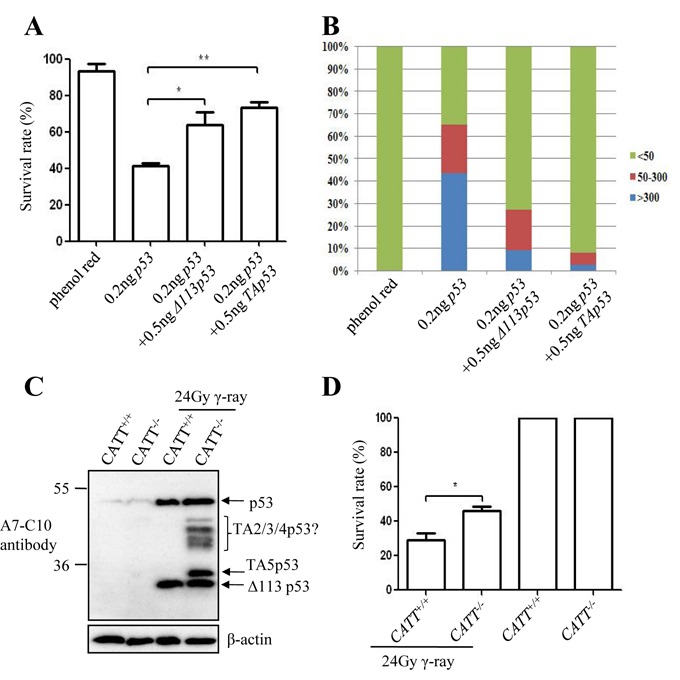
TAp53 isoforms serve as pro-survival factors by antagonizing p53 apoptotic function. (**A**) Survival rate of *tp53^M214K^* embryos injected with phenol red (control), *p53* mRNA alone, *p53* plus *Δ113p53* mRNAs and *p53* plus *345-TAp53* mRNAs. Statistic data were obtained from three independent repeats. Over 100 embryos were examined in each repeat. (**B**) Apoptosis TUNEL assay (red spots) in *tp53^M214K^* embryos at 10 hpf injected with p53 mRNA alone or co-injected with p53 and *Δ113p53* mRNA or *p53* and *345-TAp53* mRNA. Controls: buffer containing Phenol Red injected *tp53^M214K^* embryos. Statistical data were obtained from counting apoptotic cells from 30 to 50 embryos. (**C**) Western blot analysis of Δ113p53 and TAp53 isoforms in embryos 8 h after treatment with or without γ-ray. Embryos were treated with 24 Gray of γ-ray at 24 hpf. (**D**) Survival rate of embryos 5 days after being treated with γ-ray are shown. Statistical data were obtained from three batches of embryos treated with 24 Gray of γ-ray (*n* > 120 in each case). In (A) and (D), data are presented as means ± standard error. *P < 0.05, **P < 0.01.

Sequence alignment showed that all TAp53 isoforms lack the N-terminal 93 amino acids of the full-length p53 while TA2p53 added 65 extra amino acids, TA3p53 45 extra amino acids, TA4p53 18 extra amino acids and TA5p53 9 extra amino acids at their N-termini (Supplementary Figure S5). We previously showed that Δ113p53 selectively antagonizes p53 apoptotic function through Bcl-xl (9). We were intrigued to know the function of the naturally occurring TAp53 isoforms. We first injected *p53, 345-TAp53* and *289-TAp53* (with *Δ113p53* start codon ATG being mutated to ATA) mRNA, respectively, into one cell stage *p53^M214K^* (p53 null mutant) embryos and examined the expression of *p21* and *mdm2*. The result showed that the expression of *p21* and *mdm2* was robustly induced by p53 but not by TAp53 isoforms (Supplementary Figure S6A). In addition, TAp53 isoforms failed to activate the expression of *p53* (Supplementary Figure S6B). These results suggest that TAp53 isoforms alone, like Δ113p53, do not transactivate these p53-reponse genes, however, we cannot exclude the possibility that the new isoforms can transactivate other p53-repsonse genes or genes other than the ones activated by p53. Next, we co-injected *p53* mRNA with *345-TAp53* mRNA into one cell stage embryos. As expected, p53 overexpression resulted in a high rate of mortality (∼60% embryos died at 24 h post-injection) and co-injection of Δ113p53 with p53 significantly reduced the rate of mortality (less than 35% embryos died at 24 h post-injection) (Figure 5A, Supplementary Figure S7). Interestingly, we noted that TAp53 isoforms not only significantly reduced the rate of mortality caused by p53 but also appeared to be more efficient than Δ113p53 did (Figure 5A, Supplementary Figure S7). TUNEL assay revealed that these TAp53 isoforms exhibited stronger anti-apoptotic effect than Δ113p53 did on p53-mediated cell apoptosis (Figure 5B, Supplementary Figure S8). This data suggests that TAp53 isoforms also conferred anti-apoptotic function, much like the Δ113p53 isoform (9). Finally, we exposed the CATT^+/+^ and CATT^−/−^ fish to γ-ray for comparing their tolerance to the ionizing radiation treatment. Embryos at 24 hpf were irradiated with 24 Gray for 30 min and then allowed to grow in the egg water for another 5 days. We first examined the induction of Δ113p53 and TAp53 isoforms expression in CATT^+/+^ and CATT^−/−^ fish and found that, as expected, the CATT^+/+^ fish produced Δ113p53 only while the CATT^−/−^ fish produced both Δ113p53 and TAp53 isoforms (Figure 5C). The survival rate was then recorded in each case. The statistic data showed that the CATT^+/+^ fish exhibited a 29% survival rate that was significantly lower than that displayed by the CATT^−/−^ fish (46%) (Figure 5D, Supplementary Figure S9). Taken together, our results suggest that the Δ113p53 and TAp53 isoforms work together to protect embryos from the DNA damage stress.

## DISCUSSION

Genetic mutations include base substitution, deletion, inversion, duplication and crossover exchanges. Genetic mutations can be harmful, beneficial or neutral, depending on the consequence of the mutation in the genome. In this report, we show that a naturally occurring CATT 4 bp deletion in the intron 1 of the *Δ113p53* gene (also being part of the intron 4 of the *p53* gene), creates four novel p53 isoforms, namely, TA2p53, TA3p53, TA4p53 and TA5p53. We also proved that TAp53 isoforms and Δ113p53 share the same promoter and that their expressions are all induced in response to the DNA damage stress. More importantly, like Δ113p53, TAp53 isoforms function to antagonize the p53 apoptotic activity.

While p53 activation is important to eliminate DNA-damaged cells it is under strict control during early embryo development, mainly because aberrant activation of p53 is harmful to the process of embryogenesis (9,22). Therefore, how to cope with p53 activation during embryogenesis is an important biological question. In zebrafish, morpholino-mediated gene knockdown technique is routinely used in studying gene function during early development, however, it is often noted that injection of many gene-specific morpholinos resulted in off-target effect due to activation of the p53 pathway (16). Interestingly, the p53-activation-mediated off-target effect after morpholino injection on the injected embryos was eventually largely overcame in the following developmental stages (16). In addition, it is well documented that treatment with DNA-damage drug or ionization, or plasmid injection activates the p53 expression in the treated embryos (1,9,23). The availability of the monoclonal antibody A7-C10 allowed us to detect the status of p53 and Δ113p53 simultaneously under the above conditions. Using this antibody we found that embryos treated with DNA-damage drugs or γ-irradiation or the *def^hi429/hi429^* mutant embryos not only displayed an elevated level of p53 but also produced Δ113p53 and TAp53 isforms (1). The finding that both Δ113p53 and TAp53 isoforms function to antagonize p53 apoptotic activity nicely explains how nature has evolved a way to deal with DNA-damage-induced p53 activation during embryogenesis. Upon DNA-damage stress, p53 is activated which in turn activates the expression of the Δ113p53 and TAp53 isoforms. Activation of Δ113p53 and TAp53 isoforms will then protect the cells from being cleared by apoptosis and allow the cells to wait until the level of p53 is down-regulated by Mdm2. Then the cells will get a green light to re-enter the developing program. Therefore, we propose that both Δ113p53 and TAp53 isoforms are pro-survival factors. This hypothesis is supported by the fact that CATT^−/−^ fish (producing both the Δ113p53 and TAp53 isoforms) is more tolerant to ionizing radiation treatment than the CATT^+/+^ fish (producing only the Δ113p53 isoform). In this regard, the CATT 4 bp deletion is a beneficial mutation. While we showed that the CATT 4 bp deletion is beneficial to embryos upon irradiation treatment, the individual fish we genotyped was randomly picked from the population raised in our facility. We reckon that fish growing in our fish facility are under less selection pressure, together with the consideration that CATT 4 bp deletion is probably a late event in the fish population, which might explain the ratio of CATT^−/-^ observed in the population. It is not surprising that nature normally maximizes the use of the existing system to protect itself.

## ACCESSION NUMBER

p53 intron IV genomic DNA containing CATT 4bp deletion: KM981740; +32 bp transcript cDNA sequence: KM981741; +36 bp transcript cDNA sequence: KM981742; p53β cDNA sequence: KM981743.

## SUPPLEMENTARY DATA

Supplementary Data are available at NAR Online.

SUPPLEMENTARY DATA
