# Supplementary material for: A naturally occurring 4-bp deletion in the intron 4 of p53 creates a spectrum of novel p53 isoforms with anti-apoptosis function
Source: Nucleic Acids Res. 2014 Dec 29;43(2):1035–43. doi: 10.1093/nar/gku1359 (PMC4333405; doi:10.1093/nar/gku1359)
Supplement: SUPPLEMENTARY DATA [file supp_43_2_1035__index.html]

A naturally occurring 4-bp deletion in the intron 4 of p53 creates a spectrum of novel p53 isoforms with anti-apoptosis function — SUPPLEMENTARY DATA 

# A naturally occurring 4-bp deletion in the intron 4 of *p53* creates a spectrum of novel p53 isoforms with anti-apoptosis function

## SUPPLEMENTARY DATA

**Files in this Data Supplement:**

- SUPPLEMENTARY DATA
