# Supplementary material for: A naturally occurring 4-bp deletion in the intron 4 of p53 creates a spectrum of novel p53 isoforms with anti-apoptosis function
Source: Nucleic Acids Res. 2014 Dec 29;43(2):1035–43. doi: 10.1093/nar/gku1359 (PMC4333405; doi:10.1093/nar/gku1359)
Supplement: SUPPLEMENTARY DATA [file supp_gku1359_nar-02506-x-2014-File002.pdf]

```
                  *        20         *        40         *        60         *        80
hu-p53  : MEEPQSDPSVEPPLSQETFSDLWKLLPENNVLSPLPSQAMDDLMLSPDDIEQWFTEDPGPDEAPRMPEAAPPVAPAPAAPTPAAPAP
hu-p53β : MEEPQSDPSVEPPLSQETFSDLWKLLPENNVLSPLPSQAMDDLMLSPDDIEQWFTEDPGPDEAPRMPEAAPPVAPAPAAPTPAAPAP
hu-p53γ : MEEPQSDPSVEPPLSQETFSDLWKLLPENNVLSPLPSQAMDDLMLSPDDIEQWFTEDPGPDEAPRMPEAAPPVAPAPAAPTPAAPAP
zf-p53  : --MAQND-------SQE-FAELW---EKNLIIQPPGVGSCWDIIND----EEYLPGSFDPNFFENVLEEQP--QPSTLPPT------
zf-p53β : --MAQND-------SQE-FAELW---EKNLIIQPPGVGSCWDIIND----EEYLPGSFDPNFFENVLEEQP--QPSTLPPT------
              Q D       SQE F  LW     N 66 P       D66      E25      P1    6 E  P   P    PT

            *       100         *       120         *       140         *       160         *
hu-p53  : APSWPLSSSVPSQKTYQGSYGFRLGFLHSGTAKSVTCTYSPALNKMFCQLAKTCPVQLWVDSTPPPGTRVRAMAIYKQSQHMTEVVR
hu-p53β : APSWPLSSSVPSQKTYQGSYGFRLGFLHSGTAKSVTCTYSPALNKMFCQLAKTCPVQLWVDSTPPPGTRVRAMAIYKQSQHMTEVVR
hu-p53γ : APSWPLSSSVPSQKTYQGSYGFRLGFLHSGTAKSVTCTYSPALNKMFCQLAKTCPVQLWVDSTPPPGTRVRAMAIYKQSQHMTEVVR
zf-p53  : -------STVPETSDYPGDHGFRLRFPQSGTAKSVTCTYSPDLNKLFCQLAKTCPVQMVVDVAPPQGSVVRATAIYKKSEHVAEVVR
zf-p53β : -------STVPETSDYPGDHGFRLRFPQSGTAKSVTCTYSPDLNKLFCQLAKTCPVQMVVDVAPPQGSVVRATAIYKKSEHVAEVVR
                 S3VP    Y G  GFRL F  SGTAKSVTCTYSP LNK6FCQLAKTCPVQ6 VD  PP G3 VRA AIYK S2H6 EVVR

             180         *       200         *       220         *       240         *       260
hu-p53  : RCPHHERCSDSDGLAPPQHLIRVEGNLRVEYLDDRNTFRHSVVVPYEPPEVGSDCTTIHYNYMCNSSCMGGMNRRPILTIITLEDSS
hu-p53β : RCPHHERCSDSDGLAPPQHLIRVEGNLRVEYLDDRNTFRHSVVVPYEPPEVGSDCTTIHYNYMCNSSCMGGMNRRPILTIITLEDSS
hu-p53γ : RCPHHERCSDSDGLAPPQHLIRVEGNLRVEYLDDRNTFRHSVVVPYEPPEVGSDCTTIHYNYMCNSSCMGGMNRRPILTIITLEDSS
zf-p53  : RCPHHERTPDGDNLAPAGHLIRVEGNQRANYREDNITLRHSVFVPYEAPQLGAEWTTVLLNYMCNSSCMGGMNRRPILTIITLETQE
zf-p53β : RCPHHERTPDGDNLAPAGHLIRVEGNQRANYREDNITLRHSVFVPYEAPQLGAEWTTVLLNYMCNSSCMGGMNRRPILTIITLETQE
          RCPHHER  D D LAP  HLIRVEGN R  Y  D  T RHSV VPYE P26G   TT6  NYMCNSSCMGGMNRRPILTIITLE

                  *       280         *       300         *       320         *       340
hu-p53  : GNLLGRNSFEVRVCACPGRDRRTEEENLRKKGEPHHELPPGS-TKRALPNNTSS-------SPQPKKKPLDGEYFTLQIRGRERFEM
hu-p53β : GNLLGRNSFEVRVCACPGRDRRTEEENLRKKGEPHHELPPGS-TKRALPNNTSS-------SPQPKKKPLDGEYFTLQDQTSFQKEN
hu-p53γ : GNLLGRNSFEVRVCACPGRDRRTEEENLRKKGEPHHELPPGS-TKRALPNNTSS-------SPQPKKKPLDGEYFTLQMLLDLRWCY
zf-p53  : GQLLGRRSFEVRVCACPGRDRKTEESNFKKDQETKTMAKTTTGTKRSIVKESSSATSRPEGSKKAKGSSSDEEIFTLQVRGRERYEI
zf-p53β : GQLLGRRSFEVRVCACPGRDRKTEESNFKKDQETKTMAKTTTGTKRSKFAAT-----------------------------------
          G LLGR SFEVRVCACPGRDR4TEE N 4K  E        3 TKR l   3ss       s   k    d e ftlq

           *       360         *       380         *       400
hu-p53  : FRELNEALELKDAQAGKEPGGSRAHSSHLKSKKGQSTS---RHKKLMFKTEGPDSD- : 393
hu-p53β : C-------------------------------------------------------- : 341
hu-p53γ : FLINSS--------------------------------------------------- : 346
zf-p53  : LKKLNDSLELSDVVPASDAEKYRQKFMTKNKKENRESSEPKQGKKLMVKDEGRSDSD : 373
zf-p53β : --------LLKNILP------YNRRVM------------------------------ : 294
```

Fig. S1: Shi et al 2014

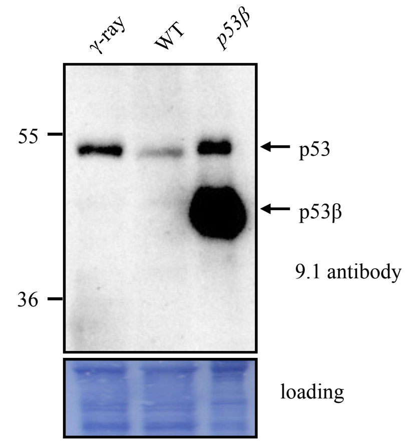

Fig. S2: Shi et al 2014

gtgagtcaaaagcgcatacacaca……gcagtgggggaggggagtgtctgcttt
tgccacagcacaaactgcagactaaaacactcAGAACAGAGATGTGTTT
CGCATTTTTAAAATATCCTGGCGAACATTTGGAGGGAGATGTT
GGTCTTTTTATGCATTTTTTAGGATGGAGTGTAATACATTTTAG
GATTGTTAATAGTGCTGGACAGTCAAGCTGGTGCTTCACATTC
TGTATGACATTACAAGACCAGGAGGGTAAGA~~CATT~~TGAAAAG
ACATTTAAAAGGCTTGAATgtaagtgtgattaaaatgtgttaattattttgt
agttcattaaattaattggtaattttgtaaatctttgtgatgatattgtcaagtgattt
atgcctgc… …tttttgtaattgcag

Fig. S3: Shi et al 2014

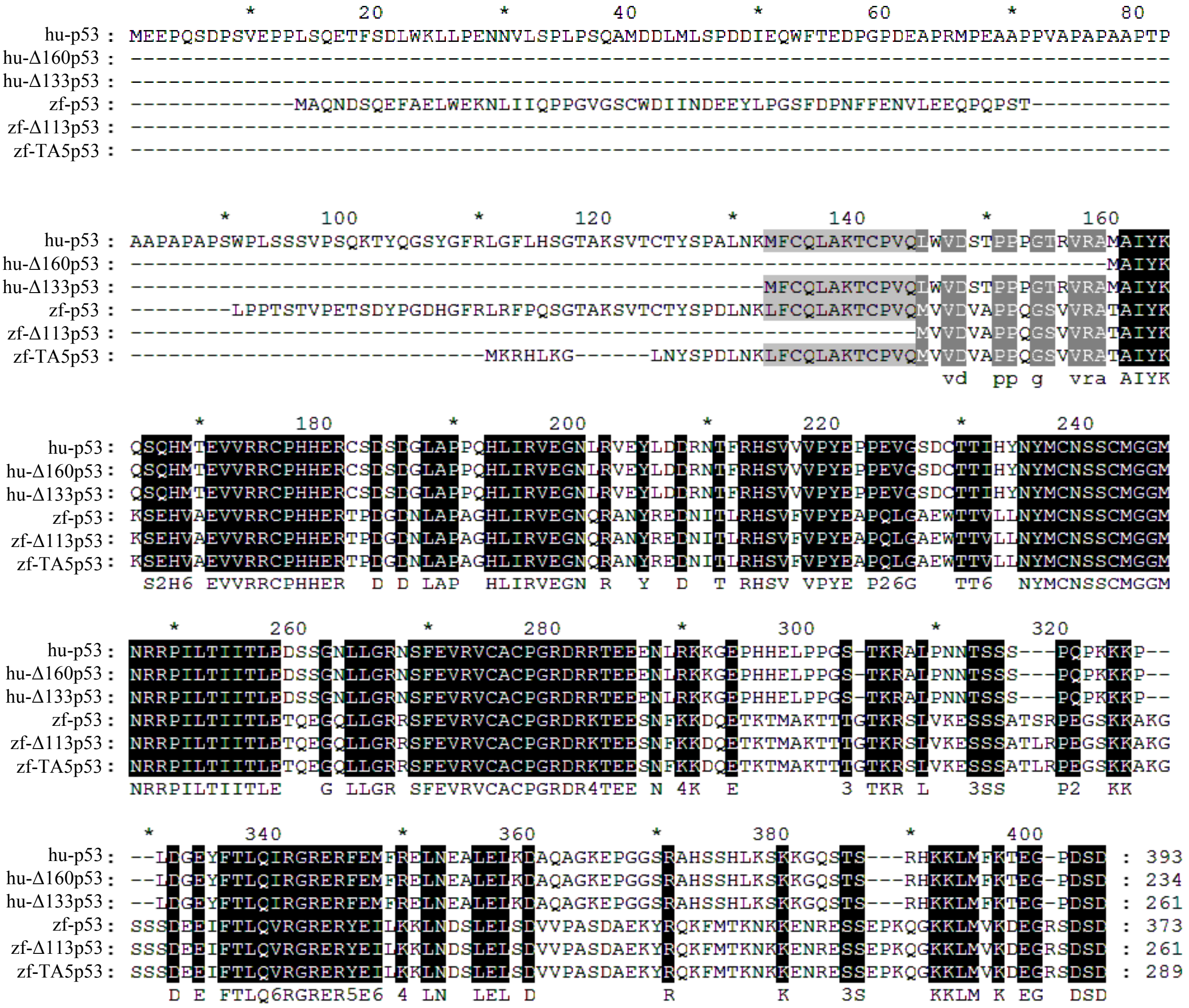

Fig. S4: Shi et al 2014

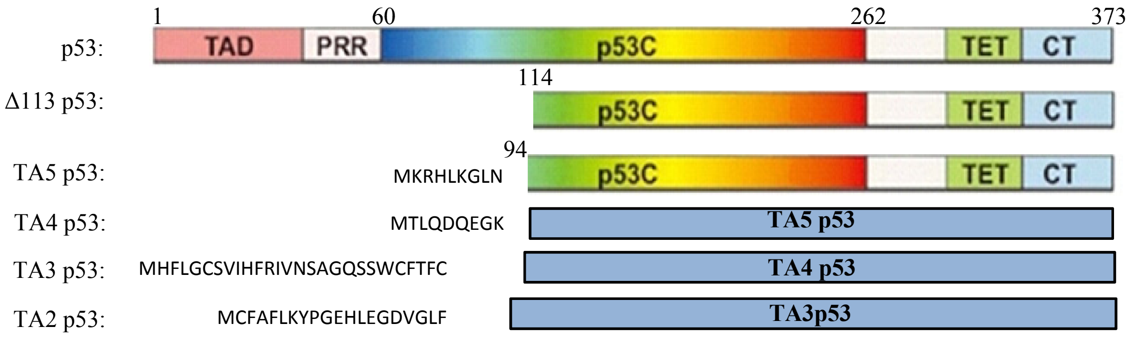

Fig. S5: Shi et al 2014

A

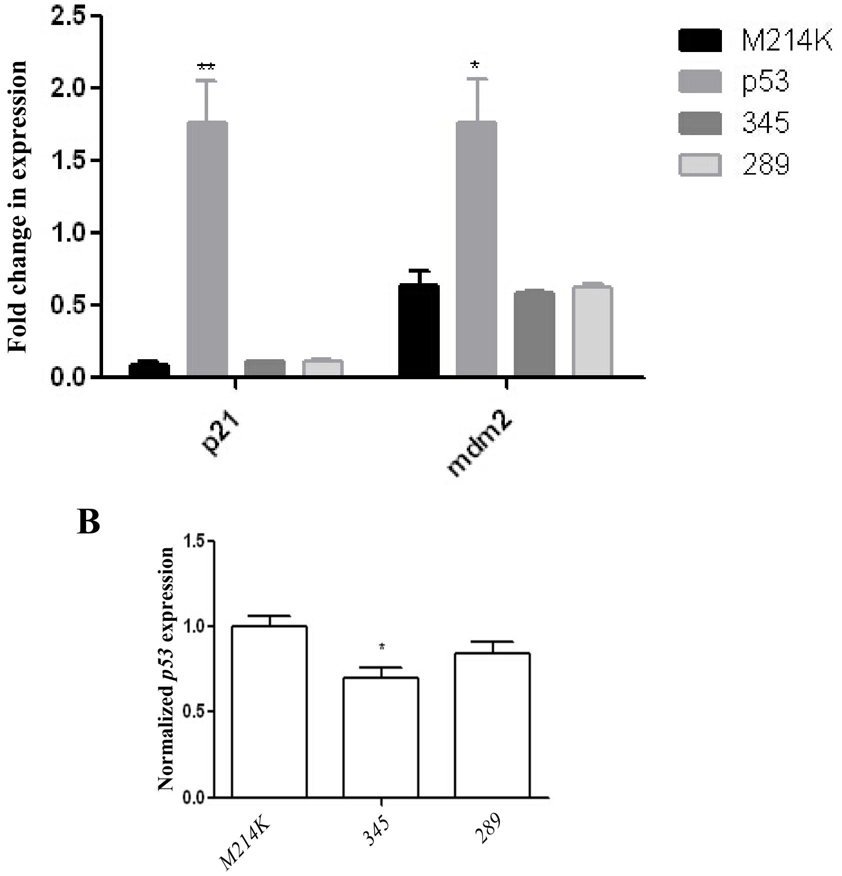

Fig. S6: Shi et al 2014

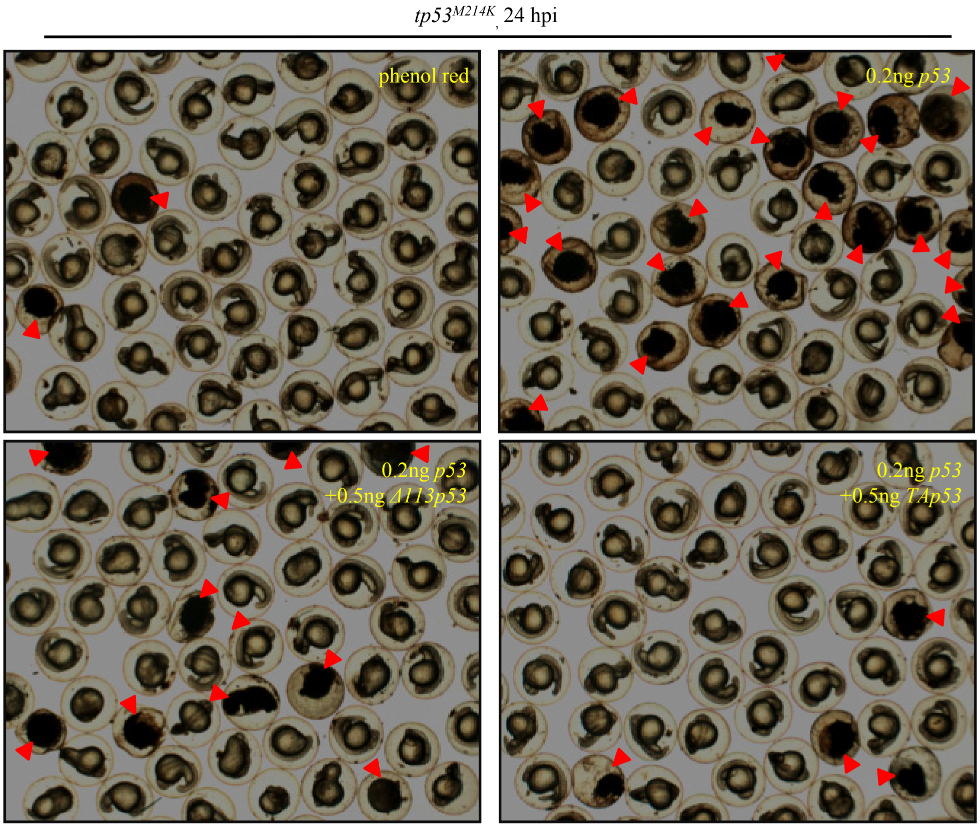

Fig. S7: Shi et al 2014

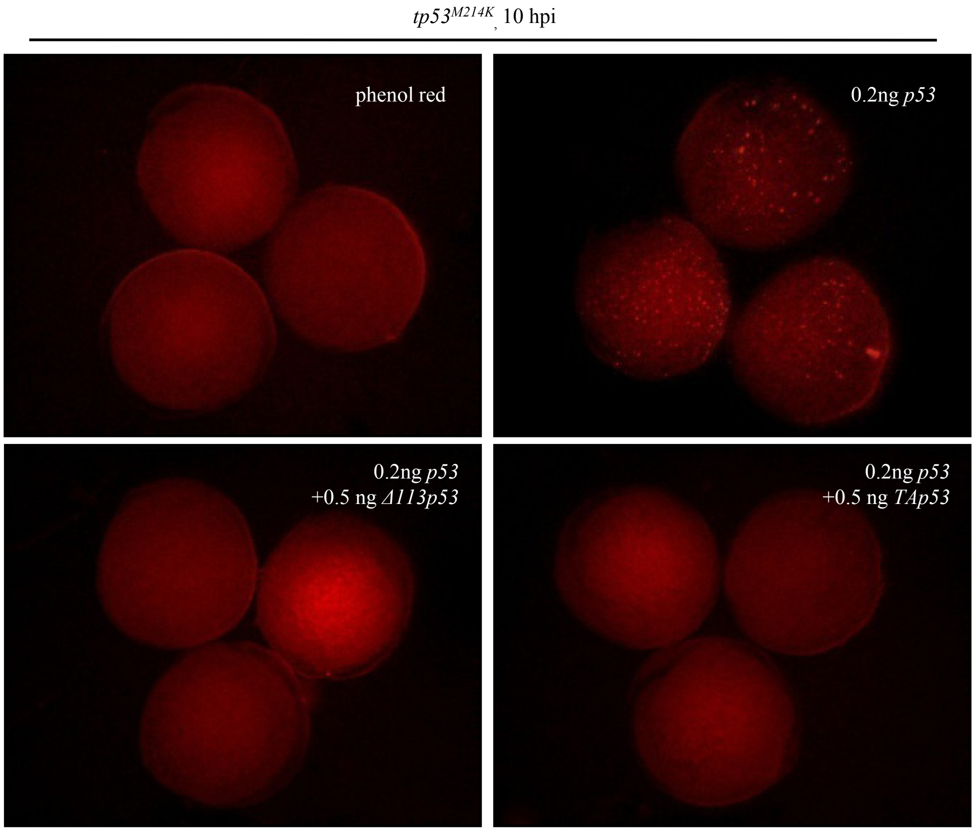

Fig. S8: Shi et al 2014

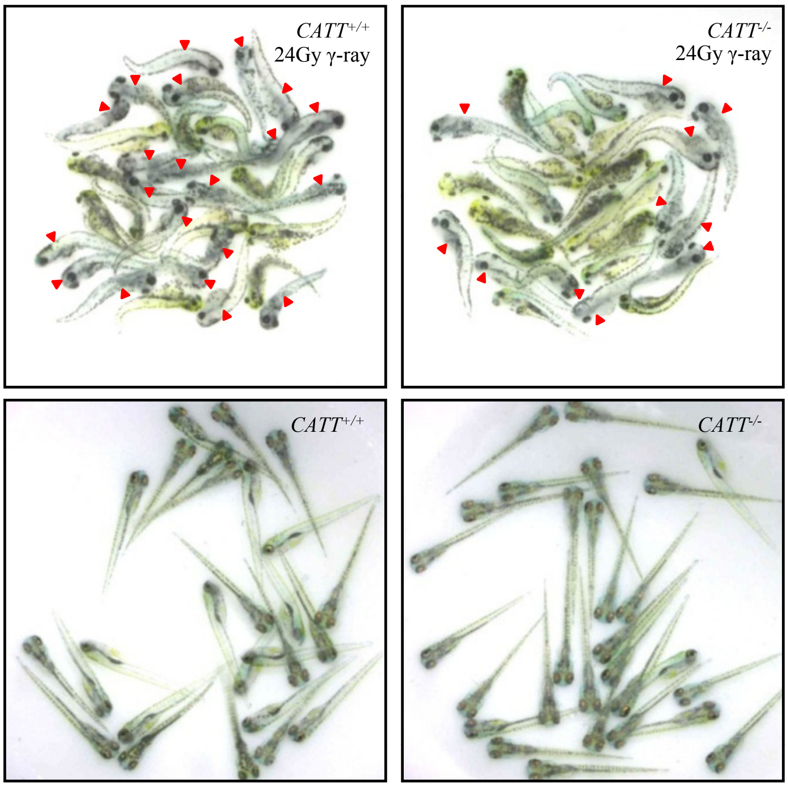

Fig. S9: Shi et al 2014

Legends for supplementary figures:

Figure S1. Alignment of amino acid sequences of human (hu-) p53, p53β, p53γ and zebrafish (zf-) p53 and p53β.

Figure S2. Western blotting to detect endogenous p53 and p53β by using the p53 antibody 9.1. The 9.1 antibody only detected p53β in the embryos injected with *p53β* mRNA (lane *p53β*) but not in WT embryos or embryos treated with γ-ray. mRNA was injected into one-cell stage embryos and total protein was extracted at 6 hpf. The γ-ray treated embryos were used as a control of p53 induction.

Figure S3. The part of the sequence of intron 4 of the *p53* gene containing the two consecutive splicing donor sites respectively for exon 1 of *Δ113p53* (blue box) and exon1 of +32/+36 transcripts (red box) is shown. Capital letters: exons, lowercase letters: flanking *p53* intron 4 sequence, four new ATG codons in +32 transcript: capital letters in red, red line crossed capital letters: CATT deletion.

Figure S4. Alignment of amino acid sequences of TA5p53 with human (hu-) p53, Δ133p53, Δ160p53 and zebrafish (zf-) p53 and Δ113p53.

Figure S5. Diagram depicting the distinct N-terminal amino acid sequences in TA2p53, TA3p53, TA4p53 and TA5p53. The accession number of the cDNA encoding for these peptides is KM981741.

Figure S6. TAp53 isoforms do not transactivate the expression of *p53* and

p53-response genes *p21* and *mdm2*. (A) Quantitative PCR (qPCR) was performed to follow the expression of *p21* and *mdm2* 6 hours after injection of *p53*, *345-TAp53* and *289-TAp53* mRNA into one cell stage *p53*^{M214K} embryos, respectively. (B) qPCR to show that the expression of p53 is not induced by overexpressing TAp53 after injection of *345-TAp53* or *289-TAp53* mRNA.

Figure S7. TAp53 isoforms function as pro-survival factors by antagonizing p53 apoptotic function. Photos showing *tp53*^{M214K} embryos injected with phenol red buffer (control), *p53* mRNA alone, *p53* plus *Δ113p53* or *p53* plus *345-TAp53* mRNA 24 hours post-injection. Dead embryos are readily identifiable by the presence of dark debris in the embryo (marked with red arrow heads).

Figure S8. TAp53 isoforms are pro-survival factors by antagonizing p53 apoptotic function. Photos showing TUNEL analysis of apoptotic cells in *tp53*^{M214K} embryos injected with phenol red buffer (control), *p53* mRNA alone, *p53* plus *Δ113p53* or *p53* plus *345-TAp53* mRNA 10 hours post-injection.

Figure S9. TAp53 isoforms are pro-survival factors by antagonizing p53 apoptotic function. Photos showing CATT$^{+/+}$ or CATT$^{-/-}$ embryos 5 days after being treated or untreated with 24 Gray of γ-ray as indicated. Surviving embryos after γ-ray treatments often exhibited a curved body. Dead embryos were readily identifiable by their gray body color (marked with red arrow heads).

Table S1. Primers used in PCR, RT-PCR, sequencing or mutagenesis

Primers	Sequence (5'-3')
**Primers (5'-3') for *p53β*cloning**	
**BamHI_*p53*_Fw**	CGCGGATCCATGGCGCAAAACGACAGCCAAGAG
**BamHI _*p53β*_ Rv**	CGCGGATCCTCACATTACCCTCCTATTATAAGG
**Primers (5'-3') for *TAp53*_345AA /325AA/298AA/289AA cloning**	
**BamHI_*TAp53*_345AA_Fw**	CGGGATCCATGTGTTTCGCATTTTTAAAATATC
**BamHI_*TAp53*_325AA_Fw**	CGGGATCCATGCATTTTTTAGGATGGAGTGTA
**BamHI_*TAp53*_298AA_Fw**	CGGGATCCATGACATTACAAGACCAGGAGGGT
**BamHI_*TAp53*_289AA_Fw**	CGGGATCCATGAAAAGACATTTAAAAGGCTTGA
**EcoRI_*p53*_Rv**	CGGAATTCTTAATCAGAGTCGCTTCTTCCTTC
**Primers (5'-3') for *TAp53* site-direct mutagenesis**	
***Δ113p53* ATG->ATA_Fw**	CCCCGTTCAAATAGTGGTGGACGTTGCCCCTCCACAGGGCTCCG TGGT
***Δ113p53* ATG->ATA_Rv**	AACGTCCACCACTATTGAACGGGGCAAGTTTTTGCCAGCTGAC AGAA
***289 AA* ATG->ATA_Fw**	ATTACAAGACCAGGAGGGTAAGATAAAAAGACATTTA
***289 AA* ATG->ATA_Rv**	ATTCAAGCCTTTTAAATGTCTTTTTATCTTACCCTCC
**Primers (5'-3') for qPCR**	
***elf1a1l*_Fw-61**	TTACCTGGCAAAGGGGAGCAGC
***elf1a1l*_Rv340**	GCACAGCACAGTCAGCCTGAGA
***zf-mdm2*_Fw620**	CTCGCAGTGAGGGCAGTGAAG
***zf-mdm2*_Rv1259**	TCTAGGCACGTAGCGGGAAGG
***zf-p21*_Fw432**	GAAGCGCAAACAGACCAACAT
***zf-p21*_Rv+484**	GCAGCTCAATTACGATAAAGA
**Other primers (5'-3')**	
***Δ113p53*_5' UTR_Fw**	CTGGCGAACATTTGGAGGGAGAT
***p53*_ExV_Rv318**	TGCCAGCTGACAGAAGAGTTTATT
***p53*_ExV_Rv351**	AACGTCCACCACCATTTGAAC
***p53*_ExV_Fw372**	GGTTCGAGCCACTGCCATCT
***p53*_ExV_Rv413**	GCCACATGCTCGGACTTCTTAT
***p53*_InVIII_Rv73**	CTGCCAGGTTAGATTCACATTA
***p53*_ExIX_Fw845**	CACGACCTGAGGGGAGCAAAAAG
***p53*_ExXI_Rv1102**	AGTCGCTTCTTCCTTCGTCCTTCA
***eGFP*_Rv22**	GCTCCTCGCCCTTGCTCAC
***eGFP*_Rv99**	CTCGCCGGACACGCTGAACTTG
